# Initial Experience of Hybrid Technique in Robot-Assisted Intracorporeal Ileal Conduit

**DOI:** 10.5152/tud.2022.22125

**Published:** 2022-11-01

**Authors:** Shugo Yajima, Yasukazu Nakanishi, Yousuke Umino, Naoya Ookubo, Kenji Tanabe, Madoka Kataoka, Hitoshi Masuda

**Affiliations:** 1 National Cancer Center Hospital East, Chiba, Japan

**Keywords:** Bladder cancer, ICUD, intracorporeal urinary diversion, RARC, robotic

## Abstract

**Objective:**

This study was designed to describe our hybrid approach to intracorporeal urinary diversion and evaluate surgical experience during initial induction.

**Material and methods:**

Clinical data from 38 patients with bladder cancer undergoing robot-assisted radical cystectomy with ileal conduit hybrid approach to intracorporeal urinary diversion at our institution between May 2020 and January 2022 were reviewed. The hybrid approach to intracorporeal urinary diversion procedure involved the following: radical cystectomy, removing a specimen through a 4- to 6-cm skin incision, harvesting an ileal conduit, redocking the robot, and uretero–uretero anastomosis. The relationship between surgical experience and operative time and a Clavien–Dindo classification of grade >3 was evaluated.

**Results:**

Of the 38 patients, 30 (79%) were male, and the median age was 75 years (interquartile range, 71-80 years). The total operative time was 384 minutes (interquartile range, 348-409 minutes). The estimated blood loss was 244 mL (interquartile range, 124-445 mL). No bowel injuries or conversions to laparoscopy or laparotomy were encountered. High-grade postoperative complications (Clavien–Dindo classification grade > 3) occurred in 7 cases (19%). The overall 90-day readmission rate following discharge after surgery was 5%. The relationship between surgical experience and operative time was nonlinear. A plateau was not reached in all 38 patients.

**Conclusion:**

Our hybrid approach to intracorporeal urinary diversion technique can be accomplished safely with acceptable operative times, even with little surgical experience. This procedure might be a safe treatment option that is relatively easy to perform, particularly in an institution that has not yet introduced intracorporeal urinary diversion. Future randomized trials with larger samples and longer follow-up periods are required to confirm our findings.

**Main Points**

- We report an overview of our hybrid approach to intracorporeal urinary diversion (h-ICUD) and its perioperative results. The h-ICUD procedure consists of radical cystectomy, removal of the specimen through an approximately 4- to 6-cm skin incision, collection of an ileal conduit, redocking the robot, and uretero–ureteral anastomosis.
- The median (interquartile range) total operative time (OT) was 384 (348-409) minutes; estimated blood loss was 244 mL (124-445 mL). There were no bowel injuries nor conversions in laparoscopy or laparotomy.
- Our h-ICUD technique can be safely accomplished with acceptable OTs, even with little experience. It appears to be a safe treatment option that is relatively easy to perform.

## Introduction

Bladder cancer is one of the most common malignancies that affect the urinary system. For muscle-invasive bladder cancer and high-risk and recurrent nonmuscle-invasive bladder cancer, radical cystectomy (RC) and urinary diversion (UD) remain standard treatments.^1^ Recently, the use of robot-assisted RC (RARC) has steadily increased worldwide, as it is a minimally invasive alternative to open or laparoscopic RC with comparable perioperative and oncological outcomes.^2^

Regarding UD following RC, because of the complex nature of the procedure, extracorporeal UD (ECUD) has traditionally been the preferred option. A new direction for reconstructive surgery is emerging with the development of robotic surgery, enabling surgeons to choose intracorporeal UD (ICUD) to complete the procedure. The advantages of ICUD include a smaller incision, less pain, and decreased bowel exposure.^3-6^ However, because of the technical complexity of the ICUD procedure and steep learning curve for surgeons, the operative time (OT) tends to be longer, particularly in cases in the early stages of implementation.^7^ Additionally, the difficulty in achieving the perfect alignment of the bowel and the risk of internal soiling with bowel contents during intracorporeal irrigation of the ileal conduit remain some of the challenges in ICUD techniques.

Incidentally, we have developed a hybrid approach to intracorporeal UD (h-ICUD), which includes a small skin incision of approximately 4-6 cm. Briefly, the procedure involves a small skin incision to remove the specimen, harvesting the ileal conduit, irrigating the ileal conduit, and then reinsufflating the abdominal cavity to perform robot-assisted UD. We believe that this h-ICUD technique has the advantage of allowing surgeons to perform sutures with precision in magnified 3-dimensional vision, using the smallest incision necessary to remove the specimen while eliminating the problems inherent in ICUD.

This study was designed to present our technique and initial experience with ileal conduit h-ICUD.

## Materials and Methods

### Patient Population and Study Design

Clinical data from 38 consecutive patients with bladder cancer undergoing RARC with ileal conduit h-ICUD at the National Cancer Center Hospital East between May 2020 and January 2022 were reviewed. Three surgeons (highly skilled and experienced) at our hospital performed ECUD until April 2020 and h-ICUD after that. We collected clinical variables, including sex, age, body mass index, Eastern Cooperative Oncology Group performance status, adjuvant chemotherapy, neoadjuvant chemotherapy, prior bacillus Calmette–Guérin (BCG) therapy, Charlson comorbidity index, OT, estimated blood loss (EBL), open conversion, complications, length of hospital stay, pathological TNM stage, and follow-up examination results. Ethical approval for this study was obtained from the Institutional Review Board of the National Cancer Center (approval number: 2018-159). All patients provided written informed consent before surgery. All procedures performed in this study followed the ethical principles of the Declaration of Helsinki.

### Surgical Technique

We performed RARC and pelvic lymph node dissection using a da Vinci XI surgical system (Intuitive Surgical Inc., Sunnyvale, Calif, USA), as follows: the abdomen was insufflated to 12 mmHg, an 8-mm camera port was then inserted in the midline, and the remaining 3 robotic ports were placed under direct vision at the level of the umbilicus in a transverse line across the abdomen. The patient was placed in the lithotomy–Trendelenburg position. A 12-mm AirSeal® (SurgiQuest Inc., Milford, Conn, USA) port and one 12-mm VersaStep™ (Covidien Inc., Mansfield, Mass, USA) were placed on the right side as assistant trocars. We began with monopolar curved scissors (Intuitive Surgical Inc.), fenestrated bipolar forceps (Intuitive Surgical Inc.), and ProGrasp™ forceps (Intuitive Surgical Inc.). The majority of the operation was performed using a 0° lens (the 30° lens was used for pelvic lymph node dissection). Then, we created the ileal conduit.

**Figure 1.**
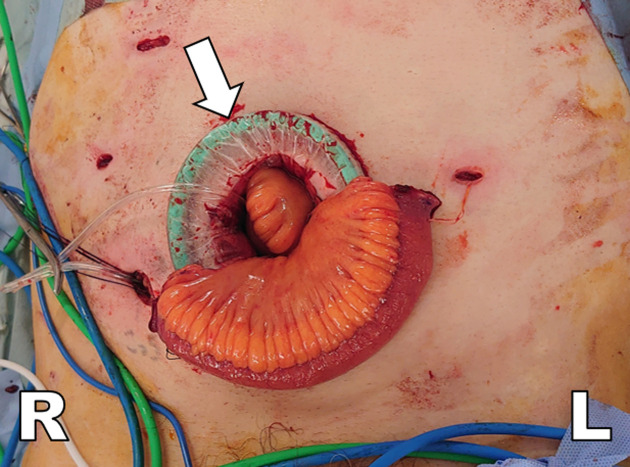
The specimen was removed from the body through an incision in the skin (approximately 4-6 cm). The skin incision was retracted circumferentially and atraumatically using a Smart Retractor® (white arrow), and then a short section of the ileum (15-20 cm) was harvested.

**Figure 2.**
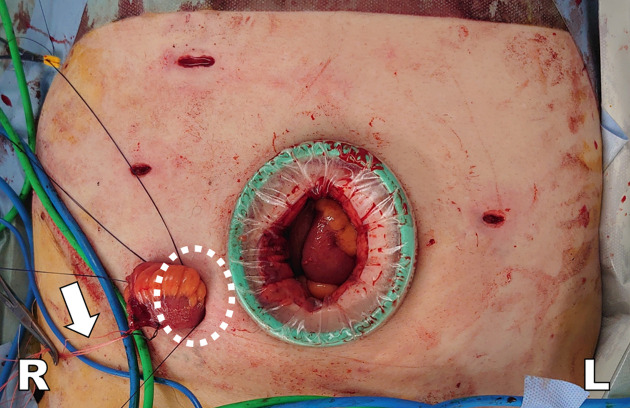
The distal end of the harvested ileum was pulled out of the abdominal wall through a small incision in the skin (stoma site, circle with white broken lines). After irrigating the ileum with saline, silk sutures were tied to the intestinal mucosa of the ileum (white arrow).

**Figure 3.**
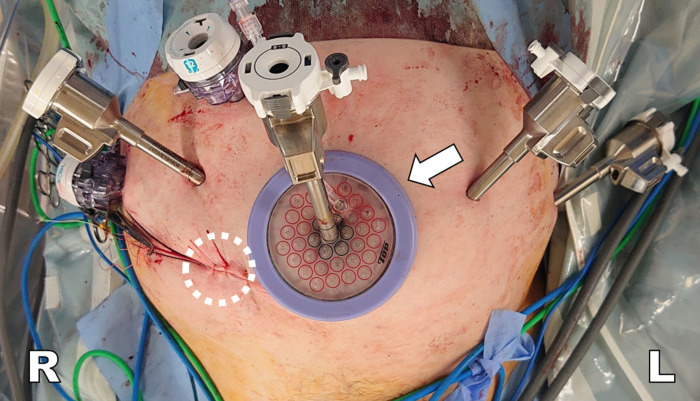
The harvested ileum was repositioned into the abdominal cavity, and the wound at the stoma site was closed with a silk suture (circle with white broken lines). The Smart Retractor® was covered with Free Access® (white arrow), and the abdominal cavity was reinsufflated.

The ileal conduit h-ICUD procedure is explained below. After completing the RARC procedure, the robot was undocked, and the patient was placed in the slight-slope Trendelenburg position. Next, the specimen was removed from the body under a minimal skin incision (approximately 4-6 cm). The skin incision was retracted circumferentially and atraumatically using a Smart Retractor® (TOP Inc., Tokyo, Japan), and a short section of the ileum (15-20 cm) was separated 20 cm proximal to the ileal valve (Figure 1). Then, the distal end of the harvested ileum was pulled out of the abdominal wall through a small skin incision (stoma site); we irrigated the ileum with saline; and we attempted using the silk suture to the intestinal mucosa of the ileum (Figure 2). The harvested ileum was then repositioned into the abdominal cavity, and the wound of the stoma site was closed using a silk suture. The Smart Retractor® was covered with Free Access® (TOP Inc.), and the abdominal cavity was reinsufflated (Figure 3). Finally, the patient was again placed in the lithotomy–Trendelenburg position, and the robot was redocked to perform uretero–uretero anastomosis, uretero–ileal anastomosis, and intracorporeal stent placement. In this procedure, at the discretion of the surgeon, the Firefly® mode was used under indocyanine green administration to check blood flow in the ureter and ileum.

**Figure 4.**
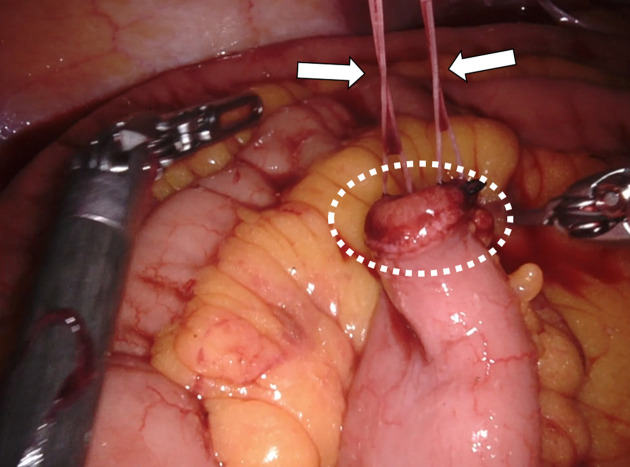
After uretero–uretero anastomosis, we pulled out the distal end of the ileal conduit (circle with white broken lines) by pulling the silk sutures (white arrow) tied to the intestinal mucosa of the ileum.

**Figure 5. a, b.**
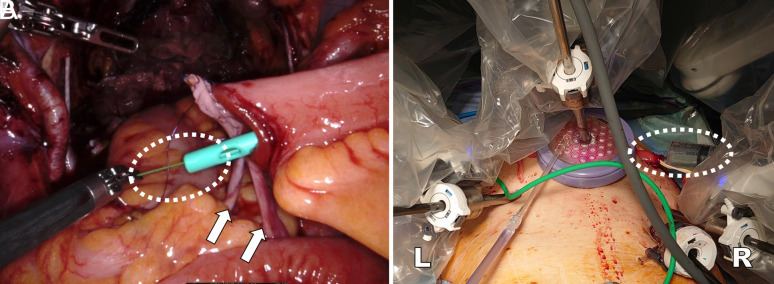
(a) Laparoscopic view. An open tip catheter with a guidewire (circle with white broken lines) was inserted in the ileal conduit to perform intracorporeal stent placement. (b) Image after bilateral ureteral stent placement. Anastomotic leak testing was performed intraoperatively (circle with white broken lines).

**Figure 6.**
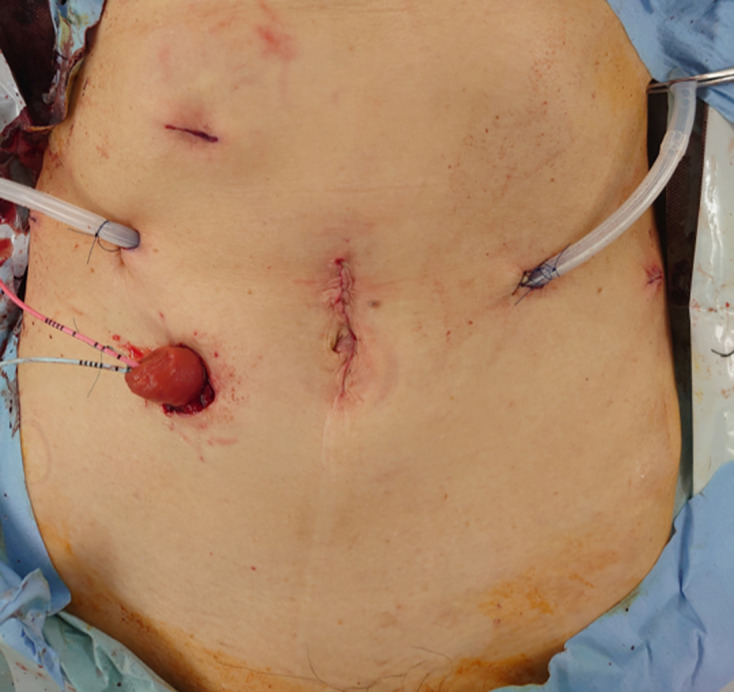
Abdominal wound findings immediately after surgery.

The intracorporeal procedure to create the uretero–uretero anastomosis was as follows: both ureters were spatulated to the same length (3-4 cm) using monopolar curved scissors, the distal and proximal ends of both spatulated ureters were marked as stay sutures using a 4‐0 polydioxanone suture, and the opposite inner borders of both ureters were over‐sewn using a running suture with a 4‐0 polydioxanone suture. Then, the free edge of the newly conjoined ureters was anastomosed to the posterior wall of an open bowel segment (proximal end of the ileal conduit) using a 4‐0 polydioxanone suture. We pulled out the distal end of the ileum through the incision at the stoma site by pulling the silk suture, which was tied to the intestinal mucosa of the ileum (Figure 4). Then, we inserted an open tip catheter (14-French scale) with 2 guidewires in the ileal conduit (Figure 5); the guidewires were inserted into both ureters. Then, 2 single‐J ureteric stents (6-French scale) were placed over the guidewires in both ureters. The conjoined ureters were anastomosed to the anterior wall of the proximal end of the ileal conduit using a 4‐0 polydioxanone suture. Finally, the robot was undocked, and the stoma was fashioned in a standard way. Figure 6 shows an image of the immediate postoperative period.

### Outcomes and Follow-Up

Postoperative complications within 90 days after surgery were recorded and scored according to the Clavien–Dindo classification (CDC).^8^ For each patient, the highest CDC grade of the most severe complication was used. Preoperative and postoperative renal function was assessed using the estimated glomerular filtration rate (eGFR), calculated using the Japanese Society of Nephrology’s equation.^9^ Obstruction at the uretero–ileal anastomotic site was diagnosed when ultrasonography or computed tomography (CT) showed dilatation of the entire upper urinary tract. Follow-up assessments were planned according to our institution’s protocol, with patients evaluated every 3 months for the first year after surgery and every 4-6 months after that. Follow-up evaluations included a physical examination, blood chemistry tests, and CT of the abdomen and pelvis.

### Statistical Analysis

Baseline demographic characteristics, perioperative data, and oncological data of the patients were analyzed using Microsoft Excel and JMP, version 13 (SAS Institute Inc., Cary, NC, USA). Continuous variables were summarized as medians and interquartile ranges (IQRs). The Shapiro–Wilk test was used to test the normality of the samples. A locally weighted scatterplot smoother (LOWESS) function^10^ was used to graphically explore the relationships between surgical experience (SE), postoperative complications, and total OT: R (version 4.1.0) was used.

## Results

### Demographics

The characteristics of the study population are presented in Table 1. Among the 38 consecutive patients who underwent RARC with ileal conduit h-ICUD at our department between May 2020 and January 2022, 30 (79%) were male. The median age was 75 years (IQR: 71-80 years); the median follow-up period was 8.5 months (IQR: 6.4-17.0 months). Preoperatively, 10 patients (26%) received BCG therapy and 17 patients (45%) received cisplatin-based neoadjuvant chemotherapy.

### Operative Characteristics and Pathological Data

**Figure 7. a, b.**
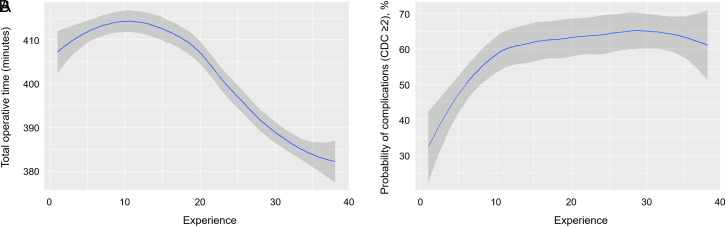
The surgical learning curve for robot-assisted radical cystectomy and intracorporeal ileal conduit (hybrid approach): effects of increasing surgical experience on total operative time (a) and the rate of postoperative complications (Clavien–Dindo grade ≥3) (b).

The operative characteristics and pathological data are presented in Table 2. The median total OT was 384 minutes (IQR: 348-409 minutes). A LOWESS function depicted a nonlinear inverse relationship between SE and total OT (Figure 7A). A plateau was not reached in all 38 patients. The median console time for RARC with pelvic lymph node dissection was 196 minutes (IQR: 173-208 minutes). The median EBL was 244 mL (IQR: 124-445 mL). No bowel injuries or conversions to laparoscopy or laparotomy were observed, and no supplementary trocars were added during the procedures. Intraoperative blood transfusion was required in 3 patients (8%); no patient needed blood transfusion postoperatively. In 1 case (3%), when Firefly® mode was used under indocyanine green administration, the blood flow of the residual ileum after harvesting ileal conduit was found to be poor, so the ileum with poor blood flow was resected and the residual ileum was re-anastomosed in a functional end-to-end manner using autosutures.

The median lymph node yield was 16 (IQR: 11-23). No patients had positive surgical margins. Pathologically positive lymph nodes were identified in 2 patients (5%).

### Postoperative Characteristics

The postoperative parameters are shown in Table 3. The median length of hospital stay was 28 days (IQR: 22-33 days). The distribution of CDC grade 2 complications was as follows: urinary tract infection in 7 cases (18%), ileus in 3 cases (8%), anemia in 2 cases (5%), and peritonitis in 1 case (3%). The relationship between SE and postoperative complications (CDC grade 3 or higher) is shown in Figure 7B. One patient had postoperative complications requiring surgery under general anesthesia (CDC grade 3b): the small bowel was strangulated by bands formed between the stump of the barbed suture and the fatty appendices of the sigmoid colon. The overall 90-day all-cause readmission rate following discharge after surgery was 5%.

After RARC with ileal conduit h-ICUD, renal function deteriorated (defined as a ≥25% decline in the eGFR at the latest follow-up) in 2 patients (5%). Obstructions at the uretero–ileal anastomotic site, diagnosed based on abdominal CT or ultrasonography, were observed in 4 cases (11%).

During the follow-up period (median: 8.5 months; IQR: 6.4-17.0 months), 6 patients (16%) experienced metastatic recurrence with a median time to progression of 4.1 months (IQR: 3.6-8.0 months); 2 (5%) patients died from tumor progression 9.0 months and 11.7 months later, respectively. No patient died from reasons unrelated to the disease.

## Discussion

Only recently has RARC with or without ICUD been explored as a viable surgical option for patients with invasive or high-risk bladder cancer. By this approach, the benefits of minimally invasive surgery are maintained and surgeons are provided with enhanced 3-dimensional surgical field visualization and operative ergonomics.^11^ A recent systematic review and meta-analysis reported that patients who underwent ICUD had significantly lower EBL and transfusion rates.^12^ However, because ICUD is a highly complex procedure, there is concern that it will result in longer OTs, an increased risk of perioperative morbidity, and a relatively slow rate of RARC–ICUD adoption.^7^ The International Robotic Cystectomy Consortium database reported that the use of ICUD increased dramatically from 9% in 2005 to 97% in 2016.^13^ However, these data from leading institutions suggest that while members of these institutions have overcome the learning curve, the outcomes do not represent the real-world application.

Several reports have compared the oncological efficacy and functional outcomes between ICUD and ECUD. By retrospectively evaluating surgical outcomes, Haber et al^14^ reported that the open-assisted laparoscopic approach was superior to the pure laparoscopic approach in OT, blood loss, transfusion rate, time to oral intake, time to ambulation, and postoperative complications (*P* < .05 for all comparisons). A large systematic review of 93 studies analyzed the perioperative outcomes and complications following RARC and demonstrated that the OT of RARC–ICUD was prolonged: the median OT for RARC with ECUD was 340 minutes (range: 292-660 minutes), while that for ICUD was 420 minutes (range: 420-450 minutes).^7^ Furthermore, this study reported that the overall 30-day complication rate following RARC with intracorporeal conduit diversion was 67% (range: 42%-86%) and 30-day high-grade complications following RARC with intracorporeal conduit diversion occurred in 24% (range: 0%-54%) of the patients. A recent meta-analysis did not show a longer OT for ICUD than that for ECUD.^12^ However, the ICUD group in the largest multicenter series of approximately 2125 patients had shorter OTs than approximately 298 patients in the 3 studies with longer ICUD OTs, suggesting the possibility of significant bias because surgeons who have already overcome the learning curve to perform ICUD. A recently published randomized controlled trial (RCT) reported a median OT of 313 minutes (IQR: 270-340 minutes) in 58 patients who underwent RARC with total ICUD, even though this cohort included 46 (79%) neobladder constructions. However, in this RCT, the median OT for the open RC group was only 190 minutes (IQR: 174-210 minutes). Therefore, the data may be significantly biased by the fact that the procedures were performed by a surgical team with sufficient experience (described in the text as having performed more than 50 procedures per year in the last 2 years prior to enrolment).^15^

Meanwhile, although the sample size in this study was small, the median total OT for RARC with an ileal conduit h-ICUD was 384 minutes (IQR: 348-409 minutes), and 7 patients (19%) had high-grade complications (CDC grade 3 or higher). The 90-day readmission rate was 5%. Considering that these initial results are from an institution that has never performed ICUD before, we believe that these data are acceptable, considering the early phase of the ICUD learning curve.^16,17^ To the best of our knowledge, this is the first study that has evaluated the short-term outcomes and effects of the learning curve on the hybrid approach to ICUD with an ileal conduit.

Our procedure has several advantages. First, extracorporeal irrigation of the ileal conduit makes it possible for surgeons to avoid internal soiling with bowel contents. Although a meta-analysis found no significant difference in overall complication rates between ECUD and ICUD and did not mention postoperative infection risk, this procedure can be performed in a shorter time, reduces intraabdominal contamination, and might reduce infectious complications. Second, by repositioning the harvested ileum into the abdominal cavity again, optimizing the mobilization of the conduit, minimizing interference, and preventing tension on the suture line in the step of the uretero–uretero anastomosis and uretero–ileal anastomosis are possible. Finally, by inserting the silicone open tip catheter into the anal side of the ileal conduit, which was pulled out of the body, intracorporeal stent placement can be performed (Figure 5). This might be simpler and less traumatic than the stenting procedure often used in ICUD, in which a suction tip is passed through the assistant port into the distal enterostomy of the ileal conduit.^17^

There are several limitations to the findings of this study. The main limitation of this study is that it is the experience of a single institution with a small series of patients without any control group, which limits the generalizability of the results. Furthermore, the follow-up periods were short, which are too short to document oncological outcomes, such as the 5-year recurrence and survival rates. We plan to present these data in the fullness of time.

In conclusion, our h-ICUD technique can be safely accomplished with acceptable OTs even with little experience. We believe that this procedure could be a safe treatment option that is relatively easy to perform, particularly in an institution that has not yet introduced ICUD. A larger series with a longer follow-up period for further assessment of long-term oncological outcomes is required to confirm our findings and the efficacy of this hybrid technique.

## Figures and Tables

**Table 1.** Characteristics of the Study Population (N = 38)

Characteristics	
Age, years, median (IQR)	75 (71-80)
BMI, kg/m^2^, median (IQR)	21.8 (19.6-23.4)
Male, n (%)	30 (79)
Charlson comorbidity index, median (IQR)	1 (0-2)
ECOG-PS, median (IQR)	0 (0-1)
Preoperative eGFR, median (IQR)	55.7 (47.0-65.0)
Prior bacillus Calmette–Guérin therapy, n (%)	10 (26)
Neoadjuvant chemotherapy, n (%)	17 (45)
Clinical T stage, n (%)	
≤T1	15 (39)
T2	14 (37)
T3	4 (11)
T4	5 (13)

BMI, body mass index; ECOG-PS, Eastern Cooperative Oncology Group performance status; eGFR, estimated glomerular filtration rate; IQR, interquartile range.

**Table 2.** Intraoperative Details and Pathological Outcomes of 38 Patients who Underwent RARC with h-ICUD

Characteristics	
Total operative time, minutes, median (IQR)	384 (348-409)
Console time for RARC, median (IQR)	196 (173-208)
Estimated blood loss, mL, median (IQR)	244 (124-445)
Conversion to open, n (%)	0
Transfusions, n (%)	3 (8)*
Positive surgical margins, n (%)	0
Lymph node yield, median (IQR)	16 (11-23)
Pathological T stage, n (%)	
T0	10 (26)
Tis	9 (24)
T1	6 (16)
T2	1 (3)
T3	6 (16)
T4	6 (16)
Pathological N stage, n (%)	
N0	36 (95)
N1	0
N2	2 (5)

h-ICUD, hybrid approach of intracorporeal urinary diversion; IQR interquartile range; RARC, robot-assisted radical cystectomy.

*Intraoperative: 3 cases, postoperative: 0 cases.

**Table 3.** Postoperative Outcomes of 38 Patients who Underwent RARC with h-ICUD

Characteristics	
Hospital length of stay, days, median (IQR)	28 (22-33)
Time to flatus, days, median (IQR)	2 (1-2)
Time to bowel, days, median (IQR)	3 (3-4)
Time to semiliquid diet, days, median (IQR)	4 (4-5)
Overall 90-day complications, n (%)	
CDC I	7 (18)
CDC II	13 (34)
CDC IIIa	6 (16)
CDC IIIb	1 (3)
CDC IV or more	0
< 90 days of readmission rate, n (%)	2 (5)
Adjuvant chemotherapy, n (%)	5 (13)
Postoperative eGFR, median (IQR)	53.1 (45.6-64.3)
Deterioration of renal function, n (%)	2 (5)
Obstruction at the uretero–ileal anastomotic site, n (%)	4 (11)

CDC, Clavien–Dindo classification); eGFR, estimated glomerular filtration rate; h-ICUD, hybrid approach of intracorporeal urinary diversion; IQR, interquartile range; RARC, robot-assisted radical cystectomy.
